# Physical properties of newly synthesized noncentrosymmetric TaIr_2_B_2_ and NbIr_2_B_2_ superconductors: an extensive comparison of GGA and LDA functional investigations†

**DOI:** 10.1039/d4ra02822h

**Published:** 2024-06-03

**Authors:** Jakiul Islam, Mohasena Ahamed, Md. Saiful Alam, Newaz Mohammad Bahadur

**Affiliations:** a Department of Physics, Noakhali Science and Technology University Noakhali 3814 Bangladesh jislam.phy@nstu.edu.bd jakiul.phy@gmail.com; b Department of Mathematics, Hajee Mohammad Danesh Science and Technology University Dinajpur Bangladesh; c Department of Applied Chemistry and Chemical Engineering, Noakhali Science and Technology University Noakhali 3814 Bangladesh; d Department of Chemistry, Noakhali Science and Technology University Noakhali 3814 Bangladesh

## Abstract

In recent years, noncentrosymmetric (NCS) structural compounds have received much attention from the scientific community in the exploration for the unconventional nature of superconductivity with exciting physical properties. This study uses the comprehensive generalized gradient approximation (GGA) and local density approximation (LDA) to gain insights into the physical properties of two recently synthesized Ir-based NCS superconductors, TaIr_2_B_2_ and NbIr_2_B_2_. The structural parameters, mechanical performance, electronic structure, Debye temperature, melting temperature, electronic specific heat, and electron–phonon coupling constant of TaIr_2_B_2_ and NbIr_2_B_2_ are explored and discussed in detail. Density functional theory (DFT) optimized structural parameters of both NCS phases agree well with experimental observation. Both GGA and LDA calculations show that the compounds are ductile, machinable, mechanically stable, and anisotropic in nature. The elastic moduli and hardness calculations reveal that TaIr_2_B_2_ is harder than NbIr_2_B_2_. The calculation of the melting temperature reveals that TaIr_2_B_2_ is more suitable for high temperature technology applications compared to NbIr_2_B_2_. Both GGA and LDA functionals reveal that the optical functions are very similar. Both compounds display a significant amount of reflectivity spectra over a wide range of photon energies. The GGA functional reveals a somewhat higher density of states value compared to that of LDA. The present calculated values of the electron–phonon coupling constant of both compounds are consistent with values previously reported from experimental studies.

## Abstract 2

In recent years, noncentrosymmetric (NCS) structural compounds have received much attention from the scientific community in the exploration for the unconventional nature of superconductivity with exciting physical properties.

## Graphical abstract

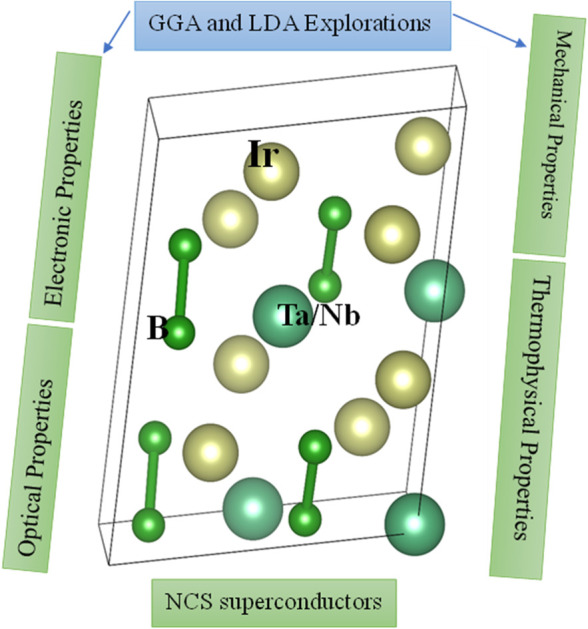

## 1. Introduction

Researchers have endeavored to discover new families of superconducting materials with novel properties since early stages of the invention of superconductivity.^1^ To scientists, it is highly challenging and fascinating to interpret the electronic configuration of a new class of superconducting compounds with diverse properties for future application in advanced quantum technologies. A recent experimental report of two Ir-based NbIr_2_B_2_ and TaIr_2_B_2_ superconductors by Górnicka *et al.*^2^ has introduced a revolutionary addition to the study of superconductivity. They revealed the noncentrosymmetric (NCS)-type structural configuration of both compounds with superconducting transition temperatures at 5.2 and 7.2 K for TaIr_2_B_2_ and NbIr_2_B_2_, respectively. Centrosymmetric superconducting compounds generally exhibit inversion symmetry, maintaining the conventional Cooper pairs. Conversely, the NCS superconductors typically do not follow the conventional Cooper pairs as a result of the lack of inversion symmetry.^2,3^ The NCS superconductors have received enormous attention by the research community due to their unconventional nature of superconductivity with fascinating physical properties.^2–13^ In 2021, Gao and his coworkers revealed the interesting topological properties in these Ir-based NCS TaIr_2_B_2_ and NbIr_2_B_2_ compounds.^4^ In the normal state, they suggested that these two Ir-based compounds are topological Weyl metals. They observed prominent topological properties such as the Weyl points, the hourglass Weyl rings, the nodal net, and the Fermi arcs close to the Fermi level. In 2022, Das and his co-authors also suggested the unconventional nature of superconductivity in NbIr_2_B_2_, along with time reversal symmetry.^5^ Due to their excellent and diverse properties, NCS superconductors have become the forefront in the field of superconductivity research. In 2022, Islam and his coworkers theoretically explored the physical and superconducting behaviours of two Rh-based NCS compounds TaRh_2_B_2_ and NbRh_2_B_2_ with chiral structure using DFT and GGA.^12^ These NCS compounds exhibited attractive properties such as their ductile nature, ease of machinability, anisotropic entity, notable values of melting temperature, and prominent reflectivity profile.^12,13^

To predict the practical application of newly synthesized materials, it is necessary to determine their fundamental physical properties, such as the mechanical performance (ductility/brittleness, hardness, and machinability), melting temperature, and optical properties (optical conductivity, reflectivity, optical absorption, dielectric function, and loss function).

An extensive literature survey reveals that the above-mentioned physical properties are still unexplored for the recently synthesized NCS TaIr_2_B_2_ and NbIr_2_B_2_ compounds. Therefore, this research deals with a detailed theoretical investigation of the physical properties of the NCS TaIr_2_B_2_ and NbIr_2_B_2_ superconductors using GGA and LDA functionals to predict the applications of these novel materials with diverse perspectives. A comparative analysis of the precision in the computational performance of these two exchange-correlation functionals GGA and LDA in investigating the properties of materials is also presented in this literature. Owing to the general affinity of GGA (LDA) for underbinding (overbinding) the crystal,^14–16^ the arithmetic averages of the GGA and LDA results of the investigated properties of the NCS compounds are also thus presented in this study to achieve a better understanding.

## 2. Methodology

In this research, the geometry optimization and calculations of the physical properties of the NCS TaIr_2_B_2_ and NbIr_2_B_2_ superconductors were executed using the DFT-based^17,18^ code Cambridge Serial Total Energy Package (CASTEP).^19^ Two well-known exchange-correlation (XC) functionals, LDA (of Ceperley and Alder (CA)^20^ – Perdew and Zunger (PZ)^21^) and GGA (of Perdew–Burke–Ernzerhof (PBE)^22^), were used in these DFT calculations. The LDA approach is assumed to be the basis of all approximated XC functionals, in which the energy is considered as a local functional (or nearly local) with regard to density. The GGA functional constructed upon LDA considers both the density, as well as its gradient. In this study, a comparative analysis between the GGA and LDA investigated properties has been presented in detail. The electron-ion interactions were performed with ultra-soft pseudopotentials (Vanderbilt-type),^23^ which uses lower energy cutoffs, along with less calculation time. The energy cutoffs of 600 eV, along with 504 irreducible *k*-points, were settled for the geometry optimization and properties calculations. The sampling of the *k*-points grid inside the first Brillouin zone was executed by inserting Monkhorst–Pack parameters.^24^ To minimize the core electron influence, pseudo-atomic calculations were treated with valence electrons only. The Broyden–Fletcher–Goldfarb–Shanno (BFGS) algorithm^25^ is inserted to ensure the optimization of the crystal's geometry. The “stress–strain” concept,^26^ along with 0.003 strain amplitude, was used to compute the elastic constants of the NCS compounds. The convergence tolerance factors for the crystal geometry optimization were parameterized as follows; energy, maximum force, maximum stress, and maximum displacement: 10^−5^ eV per atom, 0.03 eV Å^−1^, 0.05 GPa, 0.001 Å, respectively.

## 3. Results and discussion

### 3.1. Structural properties

**Fig. 1:**
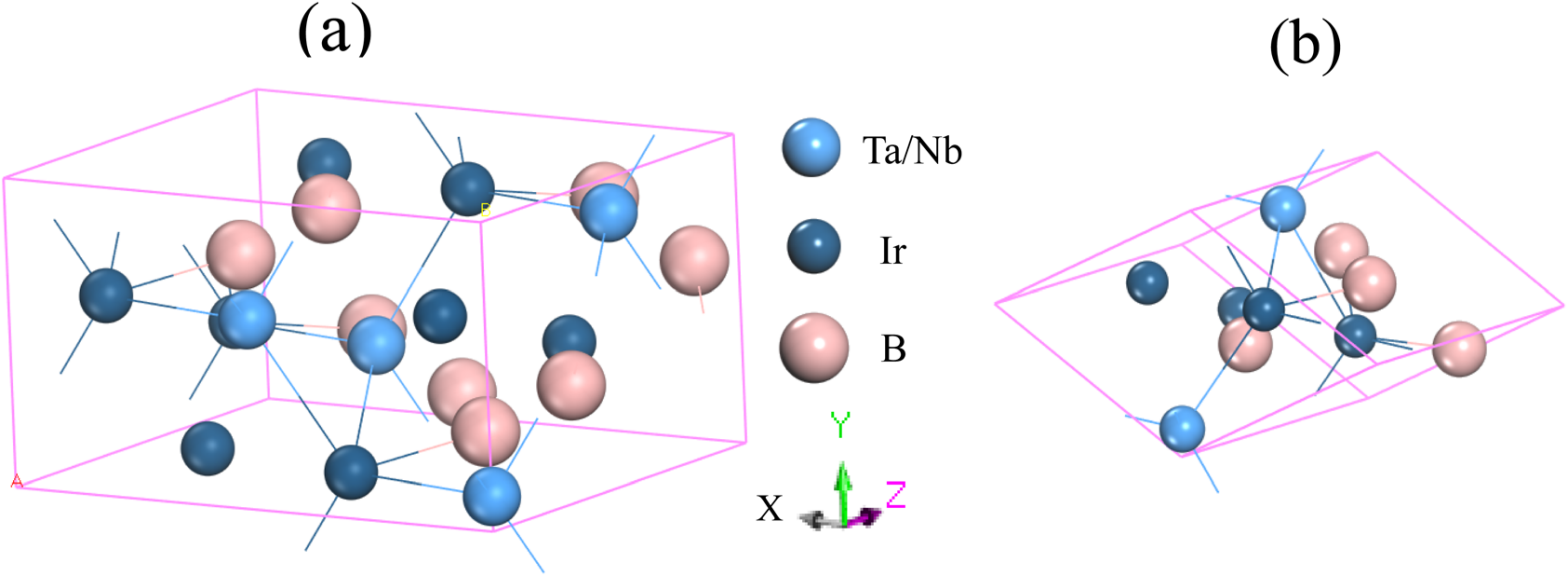
Structural configuration of the (a) conventional cell and (b) primitive cell of the TaIr_2_B_2_ and NbIr_2_B_2_ compounds.

Fig. 1 illustrates the structural configurations of the TaIr_2_B_2_ and NbIr_2_B_2_ superconductors, which possess a monoclinic system with *Cc* (no. 9) space group.^2^ The GGA-PBE and LDA-CA-PZ optimized lattice parameters of these Ir-based compounds are presented in Table 1 with the experimental report. The GGA and LDA calculated lattice parameters of the NCS compounds both show good agreement with previous experimental and theoretical reports, supporting the accuracy of this study.^2,4^Table 1 shows that the lattice constants, *a*, *b*, and *c* of GGA (LDA) are slightly larger (lower) compared to that of the experimental report,^2^ which is a familiar trend of these XC functionals.^14–16^ Consequently, the cell volume (*V*) of the GGA approach reveals a larger value than that of LDA. LDA is known to exhibit an overbinding crystal, resulting in smaller lattice constants and higher bulk moduli. Conversely, GGA tends to reveal an underbinding crystal, consequently resulting in lattice expansions (larger lattice constants) and smaller bulk moduli.^14–16^ However, the arithmetic average values of GGA and LDA of the lattice parameters are much closer to the experimental observation, as presented in Table 1. The present calculated atomic positions of these NCS compounds are summarized in Table 2, and are in good agreement with a previous analysis.^2^ The GGA-optimized values of the atomic positions of both compounds are slightly different from those of LDA.

**Table 1:** Calculated structural parameters and their deviations (Dev.) from the experimental results of the NCS TaIr_2_B_2_ and NbIr_2_B_2_ compounds

Material	Functional	*a* (Å)	Dev. (%)	*b* (Å)	Dev. (%)	*c* (Å)	Dev. (%)	*β* (°)	Dev. (%)	*V* (Å^3^)	Dev. (%)
TaIr_2_B_2_	GGA-PBE	8.2734	1.73	4.8531	1.89	6.0771	0.93	102.10	0.09	238.58	4.65
	LDA-CA	8.0783	0.67	4.7230	0.84	5.9839	0.61	102.40	0.20	222.98	2.19
	Ave.	8.1758	0.53	4.7880	0.52	6.0305	0.16	102.25	0.06	230.78	1.23
	Exp.^2^	8.1328	—	4.7631	—	6.0208	—	102.19	—	227.97	—
	GGA^4^	8.193		4.788		6.102		—			
NbIr_2_B_2_	GGA-PBE	8.2075	0.64	4.8111	0.82	6.0753	0.84	102.33	0.03	234.36	2.31
	LDA-CA	8.0753	0.97	4.7315	0.84	5.9950	0.49	102.39	0.09	223.72	2.33
	Ave.	8.1414	0.16	4.7713	0.01	6.0351	0.17	102.36	0.06	229.04	0.004
	Exp.^2^	8.1548	—	4.7718	—	6.0246	—	102.30	—	229.05	—
	GGA^4^	8.213		4.799		6.079		—	—		

**Table 2:** The present DFT-optimized atomic positions of the TaIr_2_B_2_ and NbIr_2_B_2_ compounds

Atom	TaIr_2_B_2_			NbIr_2_B_2_		
	*x*	*y*	*z*	*x*	*y*	*z*
Ta1/Nb1 (GGA)	0.00051	0.10785	0.00338	0.00945	0.11053	0.00403
Ta1/Nb1 (LDA)	0.00059	0.11016	0.00363	0.00900	0.11077	0.00355
Ir1 (GGA)	0.19293	0.61196	0.18773	0.20293	0.61353	0.18902
Ir1 (LDA)	0.19444	0.61455	0.18889	0.20323	0.61439	0.18945
Ir2 (GGA)	0.34715	0.10719	0.09714	0.35592	0.11017	0.09786
Ir2 (LDA)	0.34753	0.11210	0.09693	0.35562	0.11160	0.09728
B1 (GGA)	0.01531	0.37317	0.35376	0.02270	0.36925	0.35360
B1 (LDA)	0.01381	0.37051	0.35265	0.02259	0.37098	0.35373
B2 (GGA)	0.20167	0.19627	0.35652	0.20917	0.19340	0.35675
B2 (LDA)	0.20120	0.19210	0.35643	0.20973	0.19371	0.35726

### 3.2. Mechanical properties

The elastic parameters of the Ir-based superconductors TaIr_2_B_2_ and NbIr_2_B_2_ have not been reported yet in the previous literature to the best of our knowledge. The elastic constants are necessary to determine the mechanical stability, bonding nature, melting temperature, and anisotropy characteristics of a newly synthesized material. The monoclinic systems TaIr_2_B_2_ and NbIr_2_B_2_ have 13 independent elastic constants, *C*_11_, *C*_22_, *C*_33_, *C*_44_, *C*_55_, *C*_66_, *C*_12_, *C*_13_, *C*_23_, *C*_15_, *C*_25_, *C*_35_, and *C*_46_, as presented in Table 2. A monoclinic system is said to be mechanically stable if it justifies the following elastic stability conditions:^15^*C*_11_ > 0, *C*_22_ > 0, *C*_33_ > 0, *C*_44_ > 0, *C*_55_ > 0, *C*_66_ > 0, [*C*_11_ + *C*_22_ + *C*_33_ + 2(*C*_12_ + *C*_13_ + *C*_23_)] > 0, (*C*_33_*C*_55_ − *C*_35_^2^) > 0, (*C*_44_*C*_66_ − *C*_46_^2^) > 0, (*C*_22_ + *C*_33_ − 2*C*_23_) > 0, [*C*_22_(*C*_33_*C*_55_ − *C*_35_^2^) + 2(*C*_23_*C*_25_*C*_35_ − *C*_23_^2^*C*_55_ − *C*_25_^2^*C*_33_)] > 0, {2[*C*_15_*C*_25_(*C*_33_*C*_12_ − *C*_13_*C*_23_) + *C*_15_*C*_35_(*C*_22_*C*_13_ − *C*_12_*C*_23_) + *C*_25_*C*_35_(*C*_11_*C*_23_ − *C*_12_*C*_13_)] − [*C*_15_^2^ (C_22_*C*_33_ − *C*_23_^2^) + *C*_25_^2^(*C*_11_*C*_33_ − *C*_13_^2^) + *C*_35_^2^(*C*_11_*C*_22_ − *C*_12_^2^)] + *C*_55_(C_11_C_22_C_33_ − *C*_11_*C*_23_^2^ − *C*_22_*C*_13_^2^ − *C*_33_*C*_12_^2^ + 2*C*_12_*C*_13_*C*_23_)} > 0.

Both NCS TaIr_2_B_2_ and NbIr_2_B_2_ materials are mechanically stable, as validated by the above-written stability criteria. The dynamical stability of NbIr_2_B_2_ is observed through phonon dispersion calculation and displayed in ESI (Fig. S1†). The dynamical stability of TaIr_2_B_2_ can also be found following the similar procedure as discussed in the supplementary file. It can be seen from Table 3 that the LDA optimized values of the elastic constants of both compounds are notably higher compared to that of GGA with a few exceptions, in which the elastic constants are negative (as in *C*_15_, *C*_25_, and *C*_46_). In the case of *C*_15_, *C*_25_, and *C*_46_, the GGA and LDA functionals calculated values that are approximately close to each other. GGA provides lattice constants of higher value, and consequently provides elastic constants of lower value in comparison with LDA.^15,27^ Therefore, the arithmetic average values of the GGA-PBE and LDA-CA functionals were determined to achieve a better approximation of these elastic constants. The arithmetic average of GGA and LDA reveals that the elastic constants of three principal axes, *C*_11_, *C*_22_, and *C*_33_, are not equal to each other (*C*_11_ ≠ *C*_22_ ≠ *C*_33_), which indicates a direction-dependent behavior (anisotropic nature) among these principal axes.^28,29^ The difference between *C*_12_ and *C*_44_(*C*_12_ − *C*_44_) is noted as Cauchy Pressure (CP), which is an indicator of the ductile/brittleness property of a material.^30^ A ductile (brittle) material will have a positive (negative) CP value. Both Ir-based NCS compounds show positive CP, and hence possess ductile characteristics. This malleability nature has been clarified in this study by calculating the Poisson's ratio (*v*) and Pugh ratio (*B*/*G*). The elastic constants are necessary to determine the elastic moduli of a substance, which measure the ability of a substance to withstand applied stress. The elastic moduli are determined using the averaging schemes of Voigt–Reuss–Hill (VRH).^15^

**Table 3:** Computed elastic constants of the TaIr_2_B_2_ and NbIr_2_B_2_ compounds

Material	Functional	*C*_11_	*C*_22_	*C*_33_	*C*_*44*_	*C*_55_	*C*_66_	*C*_12_	*C*_13_	*C*_15_	*C*_23_	*C*_25_	*C*_35_	*C*_46_
TaIr_2_B_2_	GGA-PBE	499.73	475.42	472.70	65.51	151.95	142.20	182.79	228.32	−14.59	251.77	−9.49	9.56	−4.39
	LDA-CA	577.81	557.09	544.04	88.42	180.72	163.75	219.28	269.39	−14.97	288.43	−9.50	12.45	−6.49
	Ave.	538.77	516.25	508.37	76.96	166.33	152.97	201.03	248.85	−14.78	270.10	−9.49	11.00	−5.44
NbIr_2_B_2_	GGA-PBE	466.09	452.75	471.82	65.15	150.68	92.85	186.99	231.68	−15.41	250.21	−3.79	10.02	0.09
	LDA-CA	531.24	518.72	526.58	75.82	169.64	117.59	207.67	262.78	−15.28	281.08	−3.87	11.01	−1.99
	Ave.	498.66	485.73	499.20	70.48	160.16	105.22	197.33	247.23	−15.34	265.64	−3.83	10.51	−0.95

In the formulas of the elastic moduli, *B* stands for the bulk modulus, and *G* is the shear modulus. The subscripts V and R indicate Voigt and Reuss bound, respectively. The Hill approximation is indicated by the subscript H. Another elastic modulus termed as the Young's modulus (*E*) is calculated using the values of *B* and *G via* the given equation:^15^1
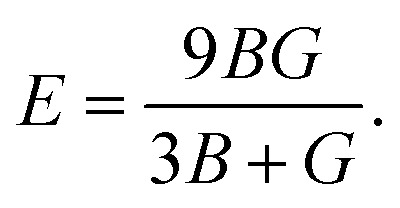


The elastic moduli *B* and *G* are also used to calculate the Poisson's ratio through the following equation:^15^2
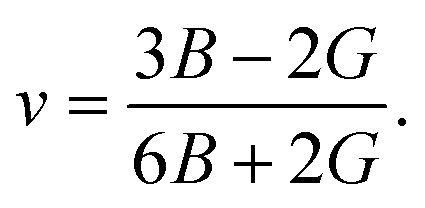


The Pugh ratio is calculated using the ratio of *B*/*G*. The obtained values of *B*, *G*, *E*, *B*/*G*, and *v* are listed in Table 4. As the GGA calculation provides elastic constants of higher value, it consequently shows elastic moduli of higher value compared to that of LDA. The bulk modulus reflects the ability of a material to resist the volume deformation (fracture) under compression. Meanwhile, the shear modulus indicates the ability to resist shear deformation (plastic deformation).

**Table 4:** Calculated elastic moduli (*B*, *G*, and *E*), compressibility (1/*B*), Poisson's ratio (*v*), machinability index (*μ*_M_), and universal anisotropy factor (*A*^U^) of the TaIr_2_B_2_ and NbIr_2_B_2_ compounds

Material	Functional	*B*_V_ (GPa)	*B*_R_ (GPa)	*B* (GPa)	1/*B* (GPa^−1^)	*G*_V_ (GPa)	*G*_R_ (GPa)	*G* (GPa)	*E* (GPa)	*B*/*G*	*v*	*μ*_M_	*A* ^U^
TaIr_2_B_2_	GGA-PBE	308.18	307.35	307.76	0.00325	124.26	111.33	117.79	313.40	2.61	0.330	4.70	0.583
	LDA-CA	359.24	358.72	358.98	0.00279	146.70	135.77	141.23	374.58	2.54	0.326	4.06	0.404
	Ave.	333.71	333.03	333.37	0.00302	135.48	123.55	129.51	343.99	2.57	0.328	4.38	0.494
NbIr_2_B_2_	GGA-PBE	303.15	301.46	302.31	0.00331	109.86	100.11	104.98	282.28	2.88	0.344	4.64	0.492
	LDA-CA	342.18	340.70	341.44	0.00293	127.61	117.13	122.37	327.93	2.79	0.340	4.50	0.452
	Ave.	322.66	321.08	321.87	0.00312	118.73	108.72	113.67	305.10	2.83	0.342	4.57	0.472

The lower (higher) value of the bulk modulus of a material indicates that it is easy (difficult) to compress. Table 4 shows that the GGA and LDA arithmetic average values of the bulk modulus are higher (lower compressibility) in TaIr_2_B_2_ compared to that of NbIr_2_B_2_. Therefore, the volume deformation of TaIr_2_B_2_ under compression is expected to be difficult compared to that of NbIr_2_B_2_. This type of behavior of the Ta and Nb analogs was also observed in previous literature studies for the Rh-based NCS compounds, TaRh_2_B_2_ and NbRh_2_B_2_.^12,13^ The Ta-analog TaIr_2_B_2_ also revealed a higher *G* compared to NbIr_2_B_2_, which indicates that the shear deformation is easier in NbIr_2_B_2_ compared to TaIr_2_B_2_. The higher *G* value of TaIr_2_B_2_ is also an indication of its higher hardness nature in comparison with NbIr_2_B_2_. For both compounds, Table 4 shows *B* > *G*, which indicates that the volume deformation is difficult in both compounds compared to the shear deformation. The ratio of *B* and *G* is also a useful parameter to determine the ductile/brittleness nature of a material. When the value of *B*/*G* is higher (lower) than the critical value of 1.75, the material is termed as having a ductile (brittle) property.^31^ Both Ir-based NCS compounds show a *B*/*G* ratio that is significantly higher than the critical value, indicating their ductile nature. The ductile nature of the Ir-based NCS compounds is also greater compared to the Rh-based NCS compounds, TaRh_2_B_2_ and NbRh_2_B_2_. NbIr_2_B_2_ shows a higher malleability nature compared to TaIr_2_B_2_ as a consequence of the lower *G* possessed by NbIr_2_B_2_ compared to TaIr_2_B_2_. The malleability nature of the Ir-based NCS compounds can be further clarified by the analysis of Poisson's ratio. The critical value of Poisson's ratio for ductile/brittleness characteristics is 0.26. A value that is higher (lower) than this indicates that the material has ductile (brittle) characteristics.^32^ The analysis of *v* also justifies the result of the Pugh ratio and CP, *i.e.*, both compounds have ductile properties. The highest values of *v* are found to be 0.344 for NbIr_2_B_2_ and 0.330 for TaIr_2_B_2_ using the GGA-PBE functional, which are somewhat higher than those of the Rh-based compounds NbRh_2_B_2_ (*v* = 0.31) and TaRh_2_B_2_ (*v* = 0.30).^12,13^

The Vickers hardness (*H*_v_) of the NCS compounds is estimated by the following model from Chen *et al.*:^33^

3 *H*_v_ = 2(*K*^2^*G*)^0.585^ − 3.

where, 
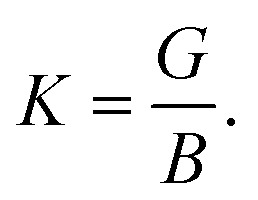


The maximum value of *H*_v_ is estimated to be 9.15 GPa for TaIr_2_B_2_ and 7.02 GPa for NbIr_2_B_2_ with the LDA functional. TaIr_2_B_2_ exhibits a higher *H*_v_ compared to NbIr_2_B_2_ as a result of the higher values of *G* occupied by TaIr_2_B_2_ in comparison with NbIr_2_B_2_.

The capability of a material to resist longitudinal stress is assessed by the value of the Young's modulus. The higher Young's modulus value of a material is also an indication of its higher stiffness. TaIr_2_B_2_ exhibits a higher value of *E* in comparison with NbIr_2_B_2_, which indicates its higher capability to withstand the longitudinal stress of the TaIr_2_B_2_ compound compared to that of NbIr_2_B_2_.

The estimation of the ease/difficulty of the device fabrication of a material is required for engineering applications, which can be known by the parameter machinability index, 
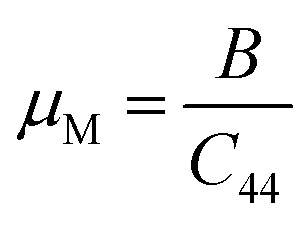
.^34^ The elastic constant *C*_44_ indicates the ability to withstand the shear deformation. The higher *μ*_M_ value of a material indicates the greater ease for fabricating it into the required shape. Generally, a hard (soft) material possesses a lower (higher) *μ*_M_ value. The hardest material diamond possesses a *μ*_M_ of about 0.8, and the softest one aluminum possesses a *μ*_M_ of about 2.6.^34^ Both studied Ir-based compounds show much higher *μ*_M_ than the softest aluminum. The machinability of these Ir-based compounds is also significantly higher compared to the Rh-based NCS compounds, TaRh_2_B_2_ (*μ*_M_ = 2.22) and NbRh_2_B_2_ (*μ*_M_ = 2.40). The GGA-PBE calculation shows that the machinability values of TaIr_2_B_2_ and NbIr_2_B_2_ are approximately close to each other. However, LDA-CA exhibits a notably higher machinability in the case of NbIr_2_B_2_ compared to TaIr_2_B_2_. The arithmetic average values of the GGA and LDA calculations of *μ*_M_ are 4.38 and 4.57 for TaIr_2_B_2_ and NbIr_2_B_2_, respectively. The higher malleability nature of NbIr_2_B_2_ (as observed from the Cauchy pressure, Pugh ratio, and Poisson's ratio) might be the result of the higher machinability of this Nb-analog compared to TaIr_2_B_2_.

The elastic anisotropy is a useful parameter for engineering applications of a material. It provides information regarding the emergence of possible microcracks in the materials under external stress. Therefore, it is very important to know the degree of anisotropy of a material before fabricating devices. The universal anisotropy factor, *A*^U^, is a well-known parameter for the analysis of the anisotropy nature. It is determined using the values of the Voigt and Reuss bulk and shear modulus *via* the following equation:^35^4
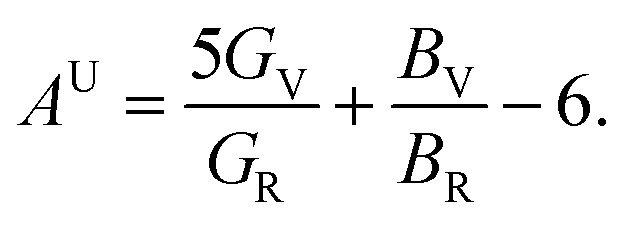


The isotropic crystal has a value of *A*^U^ = 0; any deviations from zero indicates an anisotropic nature. It can be clearly seen from Table 4 that both NCS phases have an anisotropic nature.

### 3.3. Debye temperature and melting temperature

The Debye temperature, *Θ*_D_, is a crucial thermo-physical characteristic of solids, which is closely connected with other various significant parameters, such as stiffness constants, lattice vibration, specific heat, melting temperature, thermal expansion, and superconducting transition temperature. The *Θ*_D_ defines the maximum frequency mode of the vibrations. The *Θ*_D_ can be estimated by several approaches. In this investigation, the *Θ*_D_ is determined by the use of elastic moduli. The *Θ*_D_ can be found by the following well-known expression from Anderson:^36^5
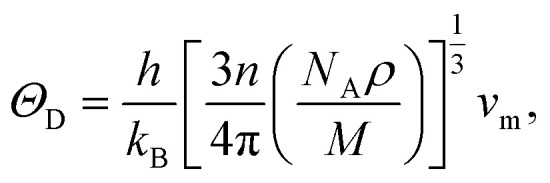
6
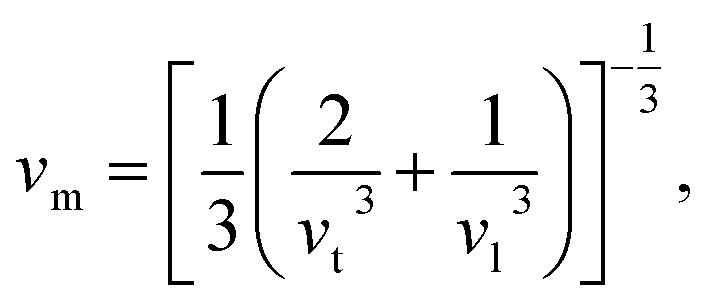
7
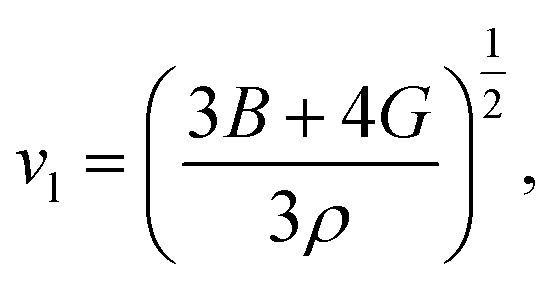
8
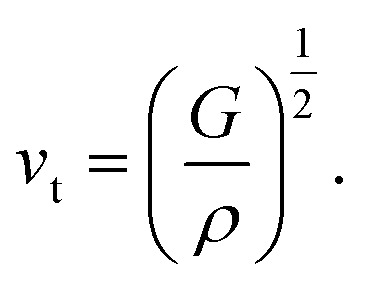
where *v*_t_, *v*_l_, *v*_m_, *h*, *n*, *N*_A_, *ρ*, *k*_B_, and *M* denote the transverse sound velocity, longitudinal sound velocity, mean sound velocity, Planck's constant, number of atoms in the molecule, Avogadro's number, crystal density, Boltzmann's constant, and molecular mass, respectively. The determined values of *v*_t_, *v*_l_, *v*_m,_*ρ*, and *Θ*_D_ are listed in Table 5. Both GGA and LDA calculations show a somewhat higher value of *Θ*_D_ for NbIr_2_B_2_ compared to that of TaIr_2_B_2_. The arithmetic average values of *Θ*_D_ are 406 and 412 K for TaIr_2_B_2_ and NbIr_2_B_2_, respectively. These values are notably higher, but follow a similar trend with the experimental observation of 230 K for TaIr_2_B_2_ and 274 K for NbIr_2_B_2_.^2^ The calculated values of hardness show that TaIr_2_B_2_ is harder in comparison with NbIr_2_B_2_. Generally, the higher hardness/stiffness material is expected to exhibit higher *Θ*_D_ than a lower hardness material.^15^ Typically, the higher hardness materials exhibit higher sound velocities as the tighter lattices propagate oscillations faster. It is interesting to note that the present study and the previous experimental observation both show a higher *Θ*_D_ value for NbIr_2_B_2_ in comparison with TaIr_2_B_2_. In previous GGA calculations, the Rh-based Nb-analog NbRh_2_B_2_ (*Θ*_D_ = 512 K) also showed a higher *Θ*_D_ value, but lower hardness compared to that of the Ta-analog TaRh_2_B_2_ (*Θ*_D_ = 480 K), which supports the present analysis.^12^ In a recent report, Hossain and his coworkers also found a higher *Θ*_D_ value (but lower hardness) in the boron-rich compound B_6_S compared to that of B_6_Se.^37^ Although the atoms involved in these materials are not that much different, their molecular masses are not the same. Moreover, their bonding distribution and variation of different elastic constants to different ranges can be the reason for the higher hardness, but lower Debye temperature.

**Table 5:** The estimated values of the crystal density (*ρ*), longitudinal sound velocity (*v*_l_), transverse sound velocity (*v*_t_), mean sound velocity (*v*_m_), Debye temperature (*Θ*_D_), melting temperature (*T*_m_), DOS at the Fermi level per formula unit, *N*(*E*_F_), Sommerfeld electronic specific heat (*γ*), and electron–phonon coupling constant (*λ*) of the TaIr_2_B_2_ and NbIr_2_B_2_ compounds

Material	Functional	*ρ* (g cm^−3^)	*v*_l_ (m s^−1^)	*v*_t_ (m s^−1^)	*v*_m_ (m s^−1^)	*Θ*_D_ (K)	*T*_m_ (K)	*N*(*E*_F_), (states per eV per f.u.)	*γ* (mJ K^−2^ mol^−1^)	*λ*
TaIr_2_B_2_	GGA-PBE	16.34	5333	2684	3009	391	2562	1.68	3.96	0.60
	LDA-CA	17.49	5787	2841	3190	425	2903	1.43	3.37	0.59
	Ave.	16.91	5560	2762	3099	408	2732	1.55	3.66	0.60
	Exp.^2^	—	—	—	—	230	—	2.06	5.2	0.70
NbIr_2_B_2_	GGA-PBE	14.14	5592	2724	3060	401	2460	1.78	4.20	0.65
	LDA-CA	14.81	5837	2874	3226	429	2737	1.60	3.78	0.64
	Ave.	14.47	5714	2799	3143	415	2598	1.69	3.99	0.65
	Exp.^2^	—	—	—	—	274	—	2.14	4.9	0.74

The melting temperature is another vital thermo-physical parameter to evaluate the applicability of a material in high temperature technology. The temperature at which a solid starts to transfer its state to the liquid phase is specified as the melting temperature (*T*_m_). *T*_m_ reflects the bond strength of a solid substance. The stiffness constants *C*_11_ and *C*_33_ are used to evaluate the *T*_m_. *C*_11_ and *C*_33_ are related to uniaxial stress. The equation of *T*_m_ is formulated as follows:^38^9
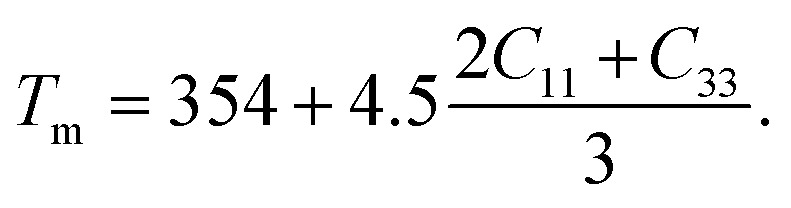


TaIr_2_B_2_ exhibits a higher *T*_m_ than NbIr_2_B_2_ because the Ta-analog possesses higher stiffness constants *C*_11_ and *C*_33_, along with a higher Young's modulus, reflecting the higher bond strength in the Ta-analog. The highest value of *T*_m_ is estimated to be 2903 K and 2737 K for TaIr_2_B_2_ and NbIr_2_B_2_, respectively, with the LDA functional. In comparison, *T*_m_ is found to be 2562 K and 2460 K for TaIr_2_B_2_ and NbIr_2_B_2_, respectively, with GGA. The *T*_m_ of these two Ir-based NCS phases are notably higher compared to that of the Rh-based NCS phases, TaRh_2_B_2_ (*T*_m_ = 2302 K) and NbRh_2_B_2_ (*T*_m_ = 2194 K).^12^ Therefore, it can be predicted that the studied Ir-based NCS phases would be much more efficient in high temperature applications than the Rh-based NCS phases TaRh_2_B_2_ and NbRh_2_B_2_. Both studied NCS phases show higher machinability along with higher *T*_m_ compared to aluminum. Generally, highly machinable materials are expected to reveal lower *T*_m_. However, the masses involved in the NCS compounds and aluminum are not the same. The bonding distribution, along with the variation of different elastic constants to different ranges, can also be the reason for such behavior. For example, we know that the machinability index is directly connected with the bulk modulus *B* and shear elastic constant *C*_44_. The variation of *B* and *C*_44_ in aluminum and the studied NCS compounds is not the same. The elastic properties of aluminum have been reported elsewhere.^39^ Further investigation on the mechanical and thermophysical properties of this type of NCS compounds can provide more clarity. For this purpose, we believe that these DFT investigations on the NCS compounds will be a useful resource to researchers in this field.

### 3.4. Electronic properties

**Fig. 2:**
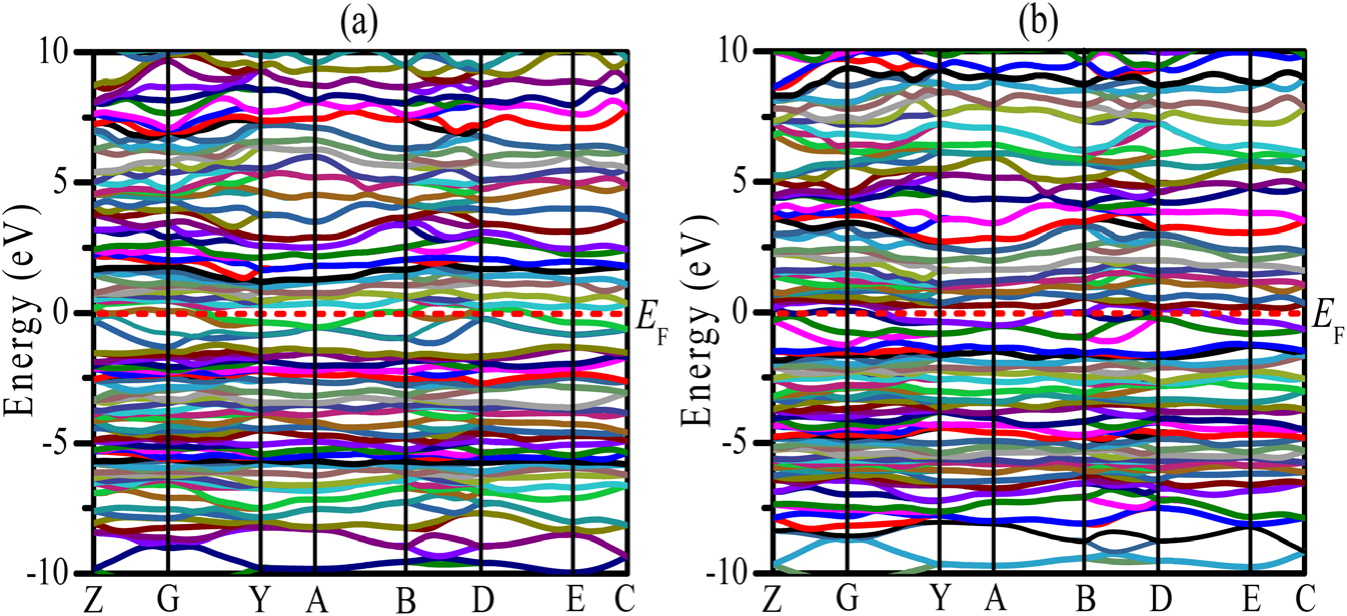
Electronic band structures of the (a) TaIr_2_B_2_ and (b) NbIr_2_B_2_ compounds using GGA-PBE.

Uncovering the technological applications of a newly synthesized material are largely dependent on the behavior of the conduction and valence electrons. Therefore, it is necessary to have a clear understanding of the electronic band structure and the density of states (DOS) of a compound. The electronic configuration of a compound relies on the energy dispersion of the valence and conduction electrons within the Brillouin zone. The calculated electronic band structures of TaIr_2_B_2_ and NbIr_2_B_2_ are presented in Fig. 2(a) and (b) using GGA-PBE towards high-symmetry directions inside the first Brillouin zone. The broken (red) line at the zero-energy scale indicates the Fermi level (*E*_F_). The energy band above (below) the *E*_F_ is the conduction band (valence band). From the band structure, it can be seen that two bands with varying levels of energy dispersion clearly cross the *E*_F_. This overlap of the valence and conduction bands at the *E*_F_ indicate the metallic character. The electronic band configurations of the Ir-based NCS superconductors with and without spin–orbit (SO) coupling were also analyzed by Górnicka *et al.*^2^ They observed the splitting of the energy bands for the addition of SO, and the deficiency of inversion symmetry.

**Fig. 3:**
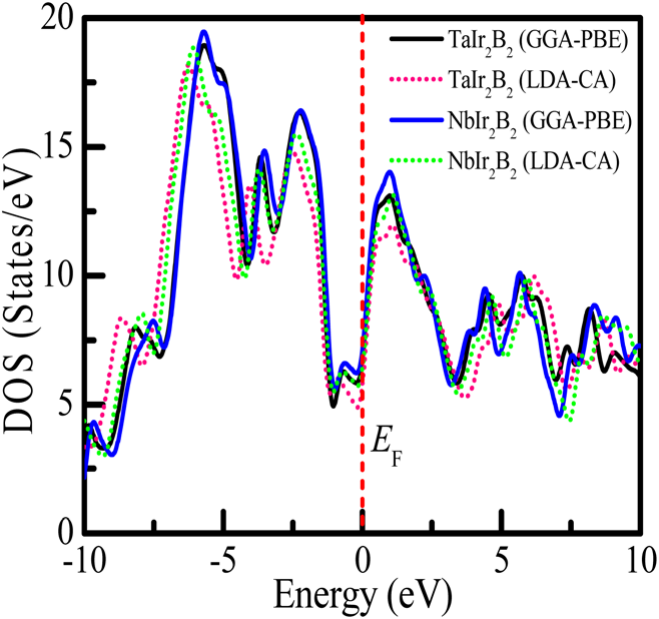
Density of states of the TaIr_2_B_2_ and NbIr_2_B_2_ compounds using GGA-PBE and LDA-CA-PZ.

The total DOS and Partial DOS of these Ir-based compounds have been analyzed to gain insight into the electronic configuration. The DOS of both NCS compounds precisely appears and is finite at *E*_F_, which is expected for a metallic system, as exhibited in Fig. 3. The values of the total DOS are found to be 1.68 states per eVper f.u. (GGA-PBE), 1.43 states per eV per f.u. (LDA-CA) for TaIr_2_B_2_, and 1.78 states per eV per f.u. (GGA-PBE), 1.60 states per eV per f.u. for NbIr_2_B_2_. This DOS analysis reveals that Nb (analog) possesses higher DOS values compared to Ta (analog). Moreover, the GGA-PBE functional provides a larger DOS value in comparison with LDA-CA. This type of behavior of these two functionals while calculating DOS was also observed in such study.^27^ However, the shape of the DOS curves is approximately the same for these two different functionals. The value of DOS (with SO) was found to be 2.06 states per eV per f.u. for TaIr_2_B_2_ and 2.14 states per eV per f.u. for NbIr_2_B_2_ in an earlier report,^2^ showing reasonable agreement with this study.

**Fig. 4:**
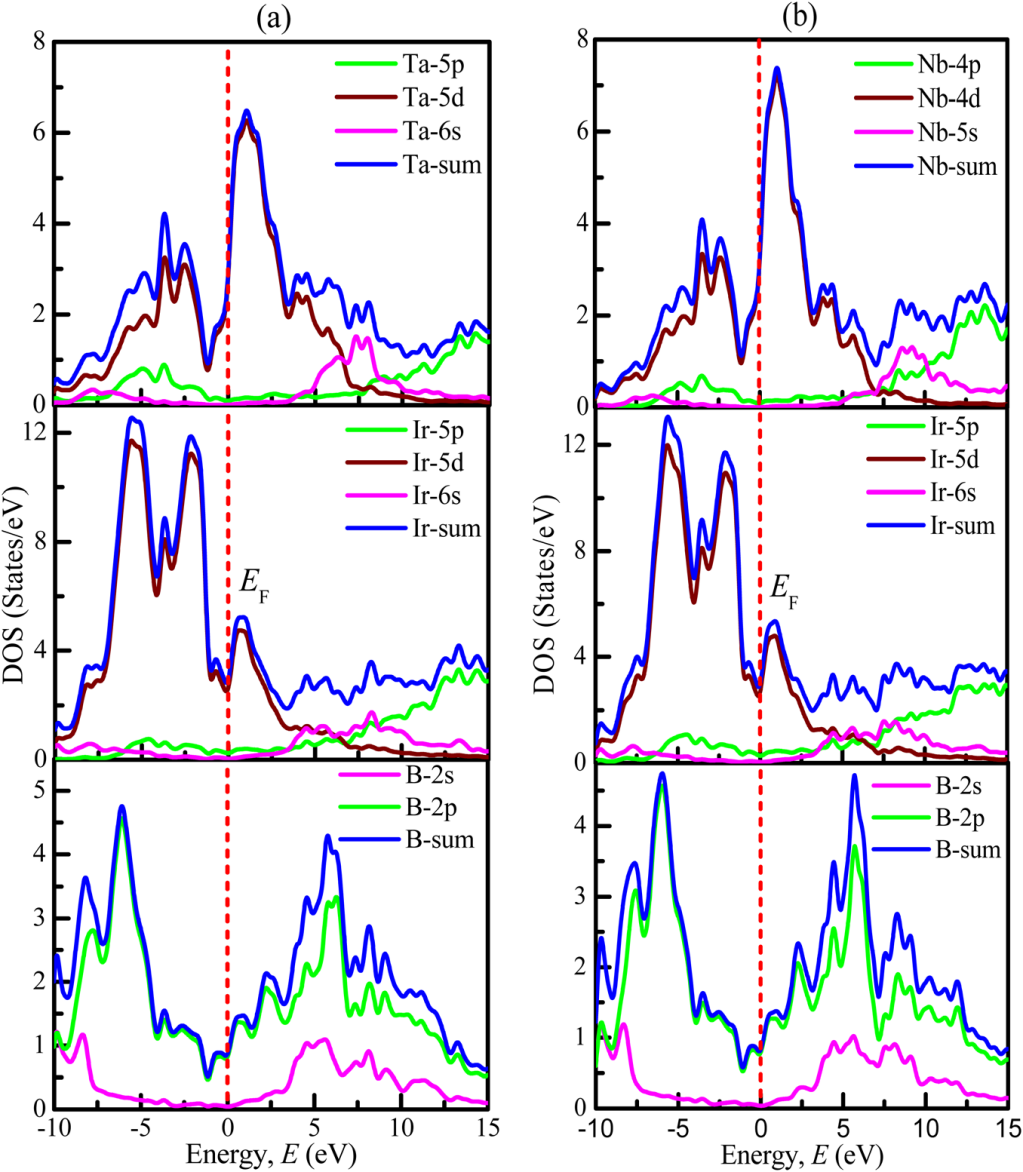
Calculated partial density of states for the (a) TaIr_2_B_2_ and (b) NbIr_2_B_2_ compounds.

The values for the partial density of states of TaIr_2_B_2_ and NbIr_2_B_2_ are calculated and displayed in Fig. 4(a) and (b), respectively. In the case of the Ta-analog, the Ta-5d, Ir-5d states are the most dominant in increasing DOS at and near *E*_F_, along with a noticeable involvement from the B-2p state. The Ta-5p, Ta-6s, Ir-5p, Ir-6s, and B-2s states reveal a negligible contribution to DOS at *E*_F_. A similar type of behavior is noticed for the Nb-analog. At and around the *E*_F_, the d-orbital contributions (Nb-4d and Ir-5d states) are the most dominant in generating DOS. The B-2p state has a significant contribution to increasing DOS at and near the *E*_F_. The overhead of the valence band (valence band near *E*_F_) is greatly dominated by the Ir-5d state for both TaIr_2_B_2_ and NbIr_2_B_2_ compounds.

### 3.5. Electronic specific heat coefficient and electron–phonon coupling constant

The Sommerfeld electronic specific heat indicator, *γ*, is estimated using the value of *N*(*E*_F_) through the following widely used equation,^40,41^10
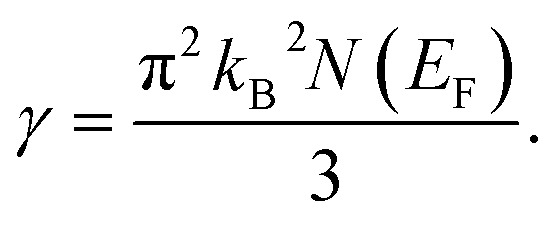


In this analysis, the GGA calculation reveals the higher value of *γ* compared to that of LDA. This is a result of the higher value of *N*(*E*_F_) in the GGA calculation than LDA. The calculated value of *γ* is estimated to be 3.96 (3.37) and 4.2 (3.78) mJ K^−2^ mol^−1^ for TaIr_2_B_2_ and NbIr_2_B_2_ using GGA (LDA), respectively.

The coupling between the electron and phonon is described by the parameter electron–phonon coupling constant, *λ*_el–ph,_ which has a crucial impact for superconducting materials. The *λ*_el–ph_ has a close relationship with the superconducting transition temperature, *T*_c_. In this study, the *λ*_el–ph_ is calculated using the value of the experimental *T*_c_, and presents the calculated Debye temperature *via* the following inverted McMillan equation,^42^11
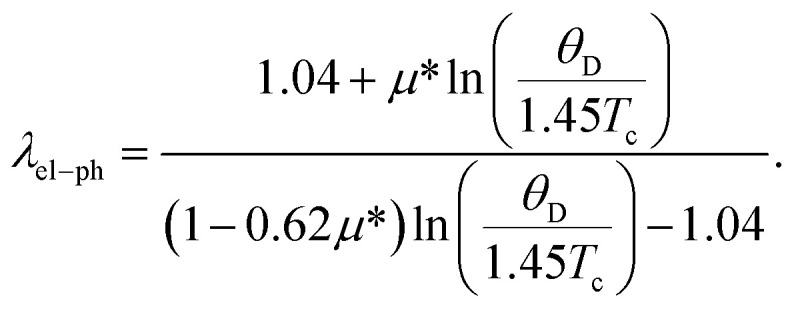
where the parameter μ* is termed as the Coulomb pseudo potential, an adjustable parameter. Typically, its value ranges between 0.1 ≤ μ* ≤ 0.15. In this calculation, the value of μ* was taken at 0.13 for evaluating *λ*_el–ph_ for both Ir-based NCS superconductors. Górnicka and his coworkers found the value of *λ*_el–ph_ = 0.70 for TaIr_2_B_2_ and *λ*_el–ph_ = 0.74 for NbIr_2_B_2_, and they recommended these Ir-based superconductors as strongly or moderately coupled superconductors. The present estimated values of *λ*_el–ph_ of both NCS superconductors are somewhat lower (presented in Table 5), but show reasonable agreement with the previous experimental report.^2^

### 3.6. Optical properties

The analysis of optical functions is very important toward determining the potential practical applications of a material. The study of optical functions is also necessary to justify the electronic structure of a newly synthesized material. The optical and electronic purpose applications of a new material can be designed through a clear understanding of the optical response of the material to photon energy. Therefore, the optical responses of TaIr_2_B_2_ and NbIr_2_B_2_ materials along different energy regions, such as infrared, visible, and ultraviolet, have been uncovered in this study using two different functionals GGA-PBE and LDA-CA-PZ. These two functionals are used to exhibit the precision of the study and the effect of the functional. This study reveals the fundamental optical parameters, such as the optical conductivity (*σ*), optical absorption (*α*), reflectivity (*R*), loss function (LF), and real (*ε*_1_) and imaginary (*ε*_2_) parts of the dielectric functions along the [100] polarization direction. A plasma energy of 5 eV along with 0.05 eV Drude damping was used during the analysis of optical functions, as a plasma energy between the scale of 2–10 eV is suggested as reasonable for a metallic system.

**Fig. 5:**
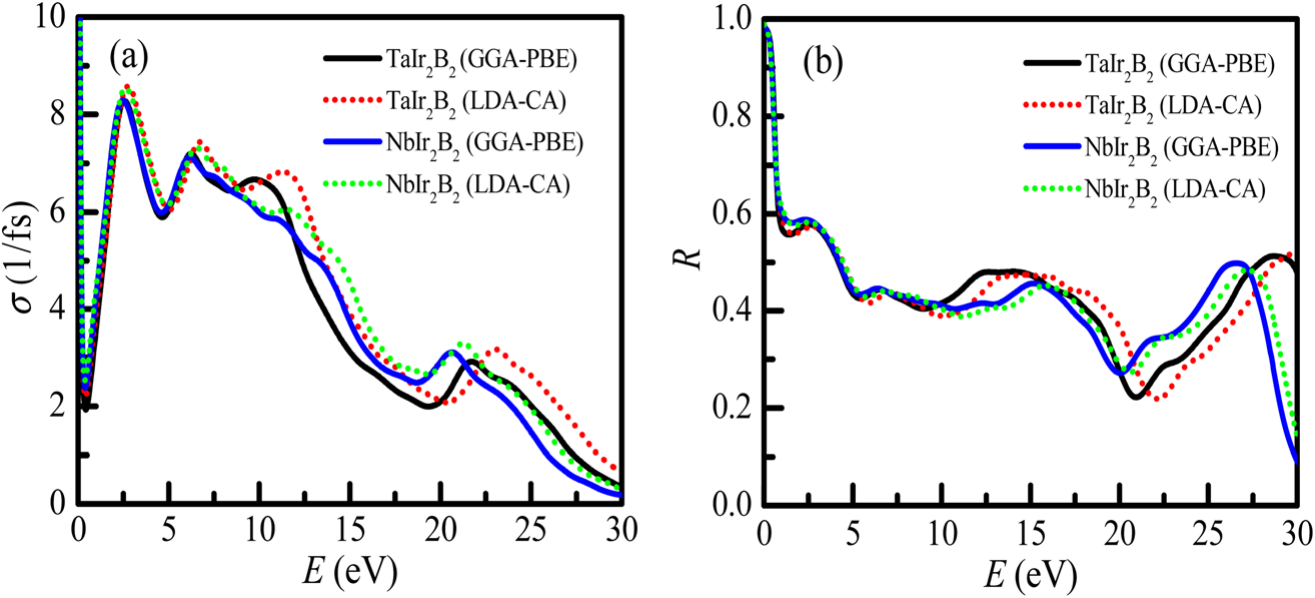
(a) Optical conductivity and (b) reflectivity of the TaIr_2_B_2_ and NbIr_2_B_2_ compounds.

The profile of the optical conductivity (real-part) of TaIr_2_B_2_ and NbIr_2_B_2_ is depicted in Fig. 5(a). The optical conductivity is related in a greater way with the photon absorption capability of a material.^27,43^ The highest peak of conductivity is observed to be at zero photon energy, an indication of a metallic system, which then abruptly decreases and further increases sharply in the visible region. A sharp notable peak of conductivity is found to be at 2.55 eV of photon energy. Then, with the increase of photon energy, the spectra of conductivity start to fall down in a regular way with few small peaks. The LDA functional shows a somewhat higher peak of conductivity spectra in the visible region, as well as in the high energy region, compared to that of GGA. However, the effect of these two XC functionals on conductivity spectra are not very notable, which is also revealed in such calculation.^27^

The reflectivity (*R*) indicates the suitability of a material to be used as a reflector. The reflectivity spectra of these compounds are depicted in Fig. 5(b), up to 30 eV of photon energy. Both Ir-based compounds show prominent reflectivity in the infrared region, as well as in the wide range of the visible energy region. The reflectivity spectra of both compounds are almost similar at up to ∼10 eV of photon energy for both GGA-PBE and LDA-CA functionals.

**Fig. 6:**
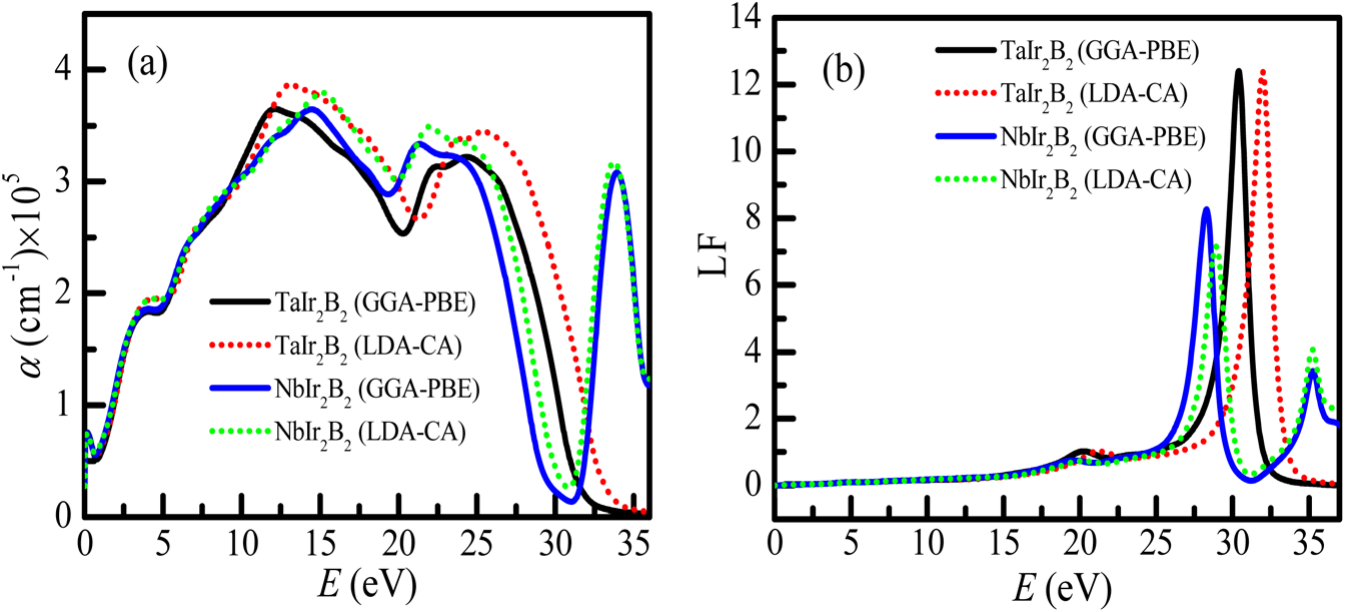
(a) Optical absorption profiles, and (b) loss function of the TaIr_2_B_2_ and NbIr_2_B_2_ compounds.

The optical absorption scheme of the Ir-based compounds is depicted in Fig. 6(a). The absorption coefficient (*α*) reflects the capability of a material to absorb photon energy while passing through it. From Fig. 6(a), it can be observed that both compounds possess no optical band gap, which reveals their metallic behavior. The absorption curves of both compounds approximately merge up to ∼11 eV. It is interesting to note that the absorption spectra of the LDA-CA functional are somewhat high above ∼12 eV compared to the GGA-PBE functional for both compounds. Both compounds show a notable optical absorption of ultra-violet (UV) energy. Hence, they can be used as UV absorbers. This type of behavior of these two functional is also observed in a previous study of the Kagome superconductor.^27^ The absorption spectra of TaIr_2_B_2_ sharply falls at ∼30 eV (plasma energy as observed from the loss function curve). Consequently, both compounds tend to be transparent to electromagnetic radiation at this energy. Such behavior is also observed in Rh-based NCS superconductors.^12,13^ However, an extra absorption peak is observed at ∼35 eV for the NbIr_2_B_2_ compound, and then the absorption spectra drop further. This extra absorption peak of NbIr_2_B_2_ might be the result of an additional peak of the loss function at around 35 eV of photon energy, as observed in Fig. 6(b).

The loss function (LF) profile of the NCS compounds is presented in Fig. 6(b). The LF provides an idea about the energy loss of high energy electrons while going through a material.^27^ These high energy electrons are attenuated and consequently generate plasma excitations (plasmons). The peak of the loss function becomes high when the energy of fast-moving electrons matches with the frequency of the plasma excitation (known as the plasma frequency). The studied NCS superconductors will act as a transparent material (metallic to dielectric-like response) above the plasma energy. For TaIr_2_B_2_, the highest peak of LF is observed at ∼30 eV (plasma energy). In the case of NbIr_2_B_2_, the sharp peak of the loss function is noticed at ∼28 eV. However, another small peak is observed at around 35 eV of photon energy in the case of NbIr_2_B_2_, which is the reason for the notable absorption peak at such high energy.

**Fig. 7:**
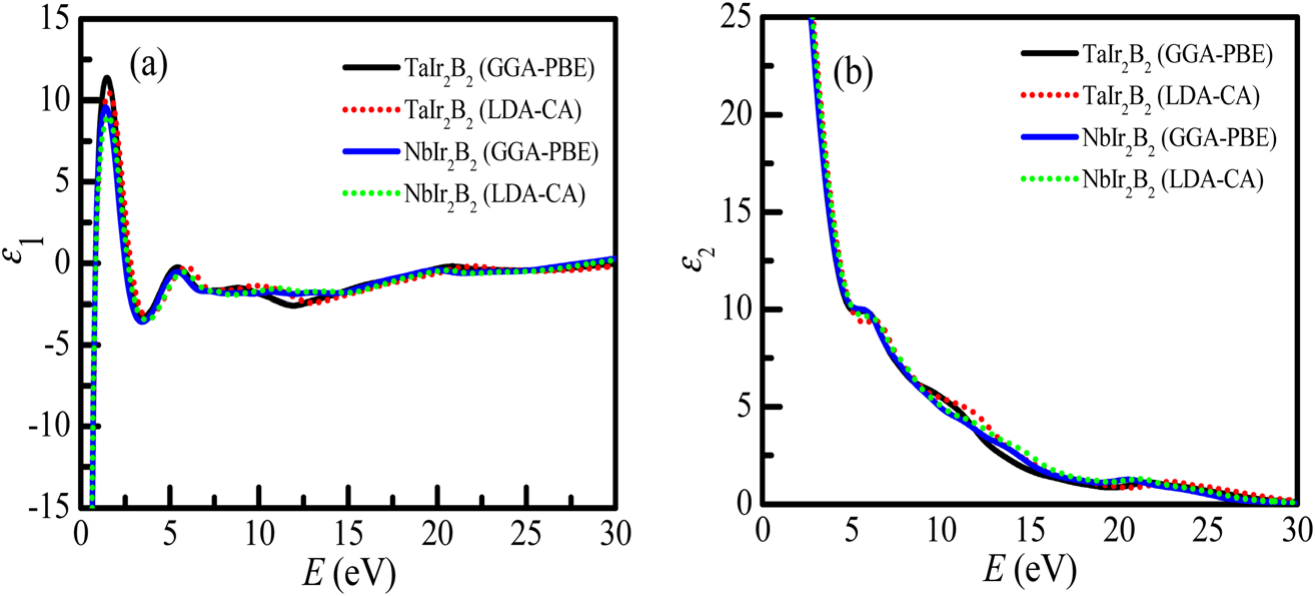
(a) Real part of the dielectric function, and (b) imaginary part of the dielectric function of the TaIr_2_B_2_ and NbIr_2_B_2_ compounds.

The real (*ε*_1_) and imaginary parts (*ε*_2_) of the dielectric function of the Ir-based NCS compounds are presented in Fig. 7a and b , respectively. The behaviors of the *ε*_1_ and *ε*_2_ spectra of the Ir-based NCS compounds reveal their metallic properties, and follow a similar trend with the Rh-based NCS superconductors TaRh_2_B_2_ and NbRh_2_B_2_ compounds.^12,13^ Both NCS phases exhibit almost similar behavior. At the low energy region, *ε*_1_ < 0, justifying the metallic property of the NCS compounds. The curve of *ε*_1_ appears sharply at 1.55 eV of photon energy. In the high energy regions, the *ε*_1_ of both NCS compounds trend to be unity, whereas the *ε*_2_ values are negligible. This behavior further reveals that both compounds might be used as transparent materials in the high energy region, as justified from the optical absorption and loss function profiles. However, in the whole energy region, the GGA-PBE and LDA-CA functionals both reveal similar spectra of the dielectric functions that is almost functionally independent.

## 4. Conclusions

The DFT calculations were performed to uncover the diverse fundamental physical properties of the Ir-based NCS compounds TaIr_2_B_2_ and NbIr_2_B_2_ using GGA and LDA functionals by running the CASTEP code. In addition, a comprehensive analysis is presented in this literature between the two GGA and LDA exchange-correlation functionals of the investigated properties. The GGA calculated values of the unit cell volume of both compounds are found to be higher compared to that of LDA, which verifies the affinity of GGA to the underbinding crystal, whereases it is overbinding in LDA. Therefore, the arithmetic averages of the GGA and LDA functionals of the lattice parameters are found to exhibit values that are much closer to the experimental observation. The calculated values of the elastic moduli using GGA are observed to be larger in comparison with the LDA calculation, which is a result of the higher value of lattice constants in the GGA calculation compared to that of LDA. Both NCS compounds justify the criteria of mechanical stability. The analysis of the Cauchy pressure, Pugh ratio and Poisson's ratio ensure that the Ir-based NCS phases TaIr_2_B_2_ and NbIr_2_B_2_ have ductile characteristics. The study of the machinability index suggests that both compounds have a significant amount of machinability. These Ir-based NCS compounds are expected to be used in fabricating devices into different desired shapes. The hardness calculations show that TaIr_2_B_2_ is comparatively harder than NbIr_2_B_2_, which is a result of the higher values of the elastic moduli possessed by TaIr_2_B_2_ compared to that of NbIr_2_B_2_. The highest value of Vicker hardness is found to be 9.15 and 7.02 GPa for TaIr_2_B_2_ and NbIr_2_B_2_, respectively, using the LDA functional. The maximum value of melting temperature is observed to be 2903 and 2737 K for TaIr_2_B_2_ and NbIr_2_B_2_, respectively, using the LDA functional. This result is a consequence of the higher stiffness constants *C*_11_ and *C*_33_, as well as the higher Young's modulus in TaIr_2_B_2_ compared to that of NbIr_2_B_2_. The higher value of melting temperature in the Ta-analog indicates its higher bond strength, along with its applicability in elevated temperature technologies. The GGA and LDA estimated values of the Debye temperature from the elastic moduli of both compounds are found to be higher compared to that of the experimental report. The maximum value of the Debye temperature is estimated to be 425 and 429 K for TaIr_2_B_2_ and NbIr_2_B_2_, respectively, with the LDA calculation. The LDA functional shows a higher Debye temperature in comparison with GGA due to the higher values of the elastic moduli in LDA compared to that of GGA. The analysis of the electronic band structure reveals no gap between the valence and conduction bands, which also justifies the analysis of optical functions. The calculated values of the electron–phonon coupling constant are found to be 0.60 (GGA) and 0.59 (LDA) for TaIr_2_B_2_, and 0.65 (GGA) and 0.64 (LDA) for NbIr_2_B_2_, which are in good agreement with the previous experimental report. The authors of this study believe that this comprehensive GGA and LDA investigation of the physical properties of NCS superconductors will attract much attention in the exploration of this new class of superconductivity.

## Conflicts of interest

The authors declare that they have no known competing financial interests or personal relationships that could have appeared to influence the work reported in this paper.

## Supplementary Material

RA-014-D4RA02822H-s001
