# Supplementary material for: Physical properties of newly synthesized noncentrosymmetric TaIr2B2 and NbIr2B2 superconductors: an extensive comparison of GGA and LDA functional investigations
Source: RSC Adv. 2024 Jun 3;14(24):17140–51. doi: 10.1039/d4ra02822h (PMC11145744; doi:10.1039/d4ra02822h)
Supplement: RA-014-D4RA02822H-s001 [file RA-014-D4RA02822H-s001.pdf]

Electronic Supplementary Material (ESI) for RSC Advances.
This journal is © The Royal Society of Chemistry 2024

Physical properties of newly synthesized noncentrosymmetric $TaIr_2B_2$ and $NbIr_2B_2$ superconductors: an extensive comparison of GGA and LDA functionals investigations

*Jakiul Islam[1], Mohasena Ahamed[2], Md. Saiful Alam[3], and Newaz Mohammad Bahadur[4]

[1]Department of Physics, Noakhali Science and Technology University, Noakhali-3814.

[2]Department of Mathematics, Hajee Mohammad Danesh Science and Technology University, Dinajpur.

[3]Department of Applied Chemistry and Chemical Engineering, Noakhali Science and Technology University, Noakhali-3814.

[4]Department of Chemistry, Noakhali Science and Technology University, Noakhali-3814.

*Corresponding author: jislam.phy@nstu.edu.bd ; jakiul.phy@gmail.com

Dynamical stability of $NbIr_2B_2$:

The dynamical stability of a compound is observed using the phonon dispersion curve. The positive frequencies in the dispersion curve implies dynamically stable material, whereas, the appearance of negative (imaginary) frequencies indicates the material as dynamically unstable [1]. The phonon dispersion of $NbIr_2B_2$ is calculated using finite displacement method [2, 3] by running CASTEP code of Materials studio-2017. The LDA functional along with OTFG ultrasoft pseudopotential was used to optimize the geometry and phonon dispersion calculation. The cutoff energy of 550 eV along with 108 irreducible k-points were used. The other convergence quality of the calculation was fixed to fine. The geometry optimization of $NbIr_2B_2$ was performed converting the crystal structure to primitive cell to minimize the computational time. The supercell defined by cutoff radius was settled to 3.5 Å during phonon dispersion calculation.

From the Fig. S1, it can be observed that only positive frequencies are presented in the phonon dispersion curve of $NbIr_2B_2$, which indicates that the compound is dynamically stable. We have demonstrated phonon dispersion for $NbIr_2B_2$ as the calculation of phonon using finite displacement method is computationally expensive and requires large computational time. Following the above-mentioned procedure, the dynamical stability of $TaIr_2B_2$ can also be observed.

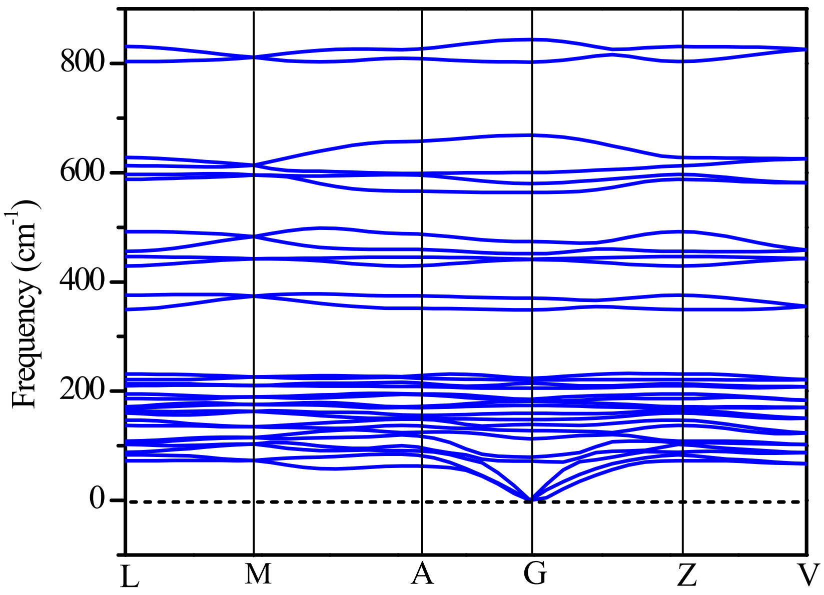

Fig. S1 Phonon dispersion curve of $NbIr_2B_2$.

References:

1. J. Islam, M.D. Islam, M.A. Ali, H. Akter, A. Hossain, M. Biswas, M.M. Hossain, M.M. Uddin, and S.H. Naqib, 2023. DFT insights into MAX phase borides Hf_2AB [A= S, Se, Te] in comparison with MAX phase carbides Hf_2AC [A= S, Se, Te], *ACS omega*, 2023, **8**, 32917-32930.
2. V. J. Minkiewicz, G. Shirane, R. Nathans, Phonon Dispersion Relation for Iron, *Phys. Rev.*, 1967, **162**, 528-531.
3. B. Montanari, N. M. Harrison, Lattice dynamics of TiO_2 rutile: influence of gradient corrections in density functional calculations, *Chem. Phys. Lett.*, 2002, **364**, 528-534.
